# Temperature- and Angle-Dependent Magnetic Properties of Ni Nanotube Arrays Fabricated by Electrodeposition in Polycarbonate Templates

**DOI:** 10.3390/nano6120231

**Published:** 2016-12-01

**Authors:** Yonghui Chen, Chen Xu, Yibo Zhou, Khan Maaz, Huijun Yao, Dan Mo, Shuangbao Lyu, Jinglai Duan, Jie Liu

**Affiliations:** 1 Institute of Modern Physics, Chinese Academy of Sciences, Lanzhou 730000, China; maaz@impcas.ac.cn (K.M.); yaohuijun@impcas.ac.cn (H.Y.); modan@impcas.ac.cn (D.M.) lvshuangbao@impcas.ac.cn (S.L.); j.duan@impcas.ac.cn (J.D.); j.liu@impcas.ac.cn (J.L.); 2 School of Physical Science and Technology, Lanzhou University, Lanzhou 730000, China; xuc13@lzu.edu.cn (C.X.); zhouyb13@lzu.edu.cn (Y.Z.); 3 Nanomaterials Research Group, Physics Division, PINSTECH, Nilore, Islamabad 45650, Pakistan

**Keywords:** Ni nanotubes, electrodeposition, polycarbonate template, magnetization reversal, angular dependent hysteresis

## Abstract

Parallel arrays of Ni nanotubes with an external diameter of 150 nm, a wall thickness of 15 nm, and a length of 1.2 ± 0.3 µm were successfully fabricated in ion-track etched polycarbonate (PC) templates by electrochemical deposition. The morphology and crystal structure of the nanotubes were characterized by scanning electron microscopy (SEM), transmission electron microscopy (TEM), and X-ray diffraction (XRD). Structural analyses indicate that Ni nanotubes have a polycrystalline structure with no preferred orientation. Angle dependent hysteresis studies at room temperature carried out by using a vibrating sample magnetometer (VSM) demonstrate a transition of magnetization between the two different magnetization reversal modes: curling rotation for small angles and coherent rotation for large angles. Furthermore, temperature dependent magnetic analyses performed with a superconducting quantum interference device (SQUID) magnetometer indicate that magnetization of the nanotubes follows modified Bloch’s law in the range 60–300 K, while the deviation of the experimental curve from this law below 60 K can be attributed to the finite size effects in the nanotubes. Finally, it was found that coercivity measured at different temperatures follows Kneller’s law within the premises of Stoner–Wohlfarth model for ferromagnetic nanostructures.

## 1. Introduction

One-dimensional nanostructures have received comprehensive attention owing to their novel optical, mechanical, catalytic, electrical, and magnetic properties and their potential applications in sensors, catalysis, field emission, and energy storage devices [1,2,3,4,5]. Nanotubes with hollow interiors have been shown to display a range of interesting properties superior to their solid counterparts [6,7]. The reason being that they possess three independent geometrical parameters for controlling their physical properties, i.e., length, *L*, and outer and inner radii, *R* and *r*. In this way, nanotubes are becoming increasingly interesting functional nanomaterials for a wide range of applications [8,9,10]. By combining the unique tubular structure with magnetic properties, magnetic nanotubes can provide an unconventional tool for their applications in biomedicine and biotechnology, such as drug delivery, targeted magnetic resonance imaging (MRI), and magneto-thermal treatment of tissues [11,12,13].

More recently, varieties of magnetic nanotubes have been prepared by different kinds of fabrication techniques [14,15,16,17,18,19,20,21,22,23]. Fe, Co, and Ni nanotubes were prepared using anodic aluminum oxide (AAO) as the host templates [14,18,21]. Fe_50_Pd_50_-based nanotubes with diameters of 200 nm and lengths of 1 µm were directly electrodeposited into polycarbonate templates [19]. Fe/Ni nanotubes with a diameter of ~110 nm were electrochemically deposited into a polyethylene-terephthalate (PET) ion-track membrane [20]. Via electroless plating on the pore's inner surface of the polycarbonate (PC) template, Ni and Co nanotubes have been fabricated via a reduction reaction on the surface of nanochannel walls [15,22,23], and similarly few other techniques have also been reported on the fabrication of magnetic nanotubes [24,25,26].

In order to realize the application of magnetic nanotubes, it is essential to understand magnetization reversal mechanisms in these nanotubes. Landeros et al. [27,28] and Escrig et al. [29,30] predicted that there are three main types of magnetization reversal modes in nanotubes: the coherent rotation mode (C), where all spins rotate simultaneously, the vortex mode (V), where the spins rotate progressively via propagation of a vortex domain wall (this mode is frequently called the curling mode), and the transverse mode (T), where the spins rotate progressively via propagation of a transverse domain wall. Different magnetization reversal modes can give different angular dependence of the coercivity, which have been thoroughly identified by numerical simulations and analytical calculations [27,29]. These magnetization reversal mechanisms have also been verified experimentally in the case of ferromagnetic nanotubes [14,19,31,32]. The transition in ferromagnetic nanotubes where the magnetization behavior changes from one reversal mode to another has been experimentally confirmed, and the transitional angle was found to depend strongly on the length, the wall thickness, and the external and internal diameters of the nanotubes [23,27,29,32]. The transition of the magnetization mode from curling to the transverse mode for Ni and Co nanotubes with small diameters has been experimentally verified [23,33]. In this paper, a different transition mechanism from curling mode to the coherent mode was observed in Ni nanotube arrays owing to the large external diameter and the thin wall thickness of these nanotubes.

Although the magnetization reversal processes in magnetic nanotubes have been studied extensively so far, there are limited reports on the study of temperature-dependent magnetic properties of the nanotubes [34,35]. Temperature-dependent magnetic studies lead to several interesting phenomena, e.g., the role of magnetization and coercivity at low temperature in the case of nanoparticles and nanowires are substantially different from their bulk cases. In bulk materials, magnetization of the system follows Bloch’s relation of the form *M*(*T*) = *M_o_*[1 − (*T/T_o_*)^α^], where α = 1.5 [36]. However, in nanowires, this law is modified to the form, where α > 1.5 (known as modified Bloch’s law), as a result of the modified spin wave structure of the system due to the finite size effects [37]. Similarly, it was found that there is a monotonic increase in coercivity with decreasing temperature, which follows the relation known as Kneller’s law of the form *H_c_ = H_o_*[1 − *T/T_B_*]^1/2^ [38,39], where *H_o_* is the coercivity at *T* = 0 K, and *T_B_* is the blocking temperature of the system. At very low temperatures, the deviation of *H_c_* from the above relation was explained as a result of the surface spins which freeze in their random states, giving no further response to the applied magnetic field that resultantly displays a deviation of the experimental curve from Kneller’s law [40]. A similar trend is also possible in the case of magnetic nanotubes.

In this work, Ni nanotube arrays were fabricated using a nanoporous polycarbonate membrane via an electrochemcial deposition technique. The structure and morphology of the prepared Ni nanotubes were characterized by scanning electron microscopy (SEM), transmission electron microscopy (TEM), and X-ray diffraction (XRD), respectively. The angular dependence of coercivity and remanence for Ni nanotube arrays were investigated via a vibrating sample magnetometer (VSM), while magnetic properties at different temperatures ranging from 5 K to 300 K were measured via a superconducting quantum interference device (SQUID). The aim of this research was to present a systematic study of the magnetic characterization of Ni nanotubes including angle- and temperature-dependent magnetic properties and to tune and tailor these properties for possible technological applications of these nanotubes.

## 2. Materials and Methods

Polycarbonate membranes with 30 µm thicknesses were first irradiated at the Heavy Ion Research Facility in Lanzhou (HIRFL, Lanzhou, China) with ^209^Bi ions with an initial kinetic energy of 9.5 MeV/u and a fluence of 5 × 10^8^ ions/cm^2^. In the second step, the membranes were etched in a 5 M NaOH solution at 50 °C for 5 min to obtain cylindrical nanopores with diameters of about 150 nm. Prior to deposition, a thin Au film (~60 nm) was sputtered onto one side of the membrane and served as a conducting substrate (acted as cathode) for the deposition of nanotubes. This method has been previously reported for nanotubes prepared via potentiostatic deposition in an AAO template [14,21]. In order to form the nanotubes, a relatively large voltage of 2.0 V (nanowires were produced blow this voltage) was potentiostatically performed during Ni deposition in a two-electrode cell (with Ni as the anode) using the electrolyte as the mixture of 250 g/L NiSO_4_·6H_2_O, 50 g/L NiCl_2_·6H_2_O, and 30 g/L H_3_BO_3_. Electrodeposition was carried out for a short period of time (~3 min) at room temperature.

After dissolving the PC templates in dichloromethane (CH_2_Cl_2_), the morphology and structures of the nanotubes were investigated via scanning electron microscopy (SEM, Nova NanoSEM 450, FEI Company, Eindhoven, The Netherlands) and transmission electron microscopy (TEM, Tecnai G2 F20, FEI Company, Eindhoven, The Netherlands). The crystalline textures and magnetic properties of the nanotubes were analyzed via X-ray diffraction (XRD, X’Pert PRO, PANalytical, Almelo, The Netherlands, Cu with Kα of λ = 0.154056 nm), a vibrating sample magnetometer (VSM, EV9, MicroSense, Lowell, MA, USA), and a superconducting quantum interference device (SQUID, MPMS-XL, Quantum Design, San Diego, CA, USA), respectively. For magnetic analyses, the nanotubes were kept embedded in the host templates.

## 3. Results and Discussion

**Figure 1:**
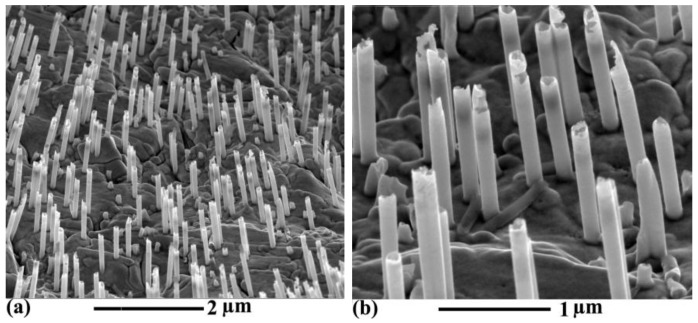

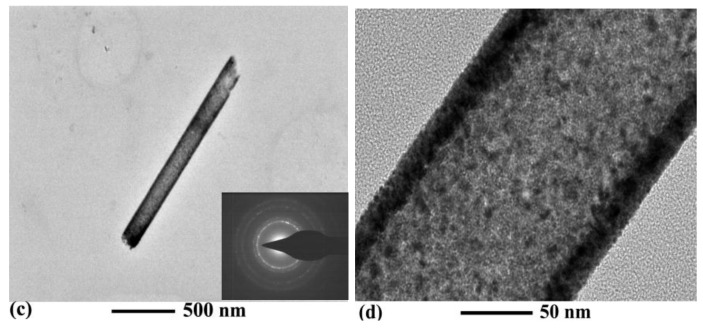
Scanning electron microscopy (SEM) images of Ni nanotube arrays with (**a**) low magnification and (**b**) high magnification; Transmission electron microscopy (TEM) images of a single Ni nanotube with (**c**) low magnification and (**d**) high magnification, showing an outer diameter of 150 nm, a wall thickness of 15 nm, and a length of 1.2 ± 0.3 µm. The inset shows the corresponding selected area electron diffraction (SAED) pattern.

Figure 1 shows the SEM and TEM images of Ni nanotubes. In Figure 1a,b, the free-standing Ni nanotube arrays after dissolving the PC template are displayed. It is seen that the nanotubes are almost parallel to each other and uniformly distributed on the substrate. Figure 1c,d shows the TEM images of a single Ni nanotube liberated from the template. It is apparent that the Ni nanotube has a uniform wall thickness of about 15 nm and a length of 1.2 ± 0.3 µm. The error in the length results from the statistical measurement of multi samples of a single Ni nanotube. The average outer diameter of the nanotubes is about 150 nm that corresponds to the diameter of the nanochannels in the PC template after the etching process. The inset of Figure 1c represents the selected area electron diffraction (SAED) pattern, which indicates the polycrystalline nature of Ni nanotubes.

**Figure 2:**
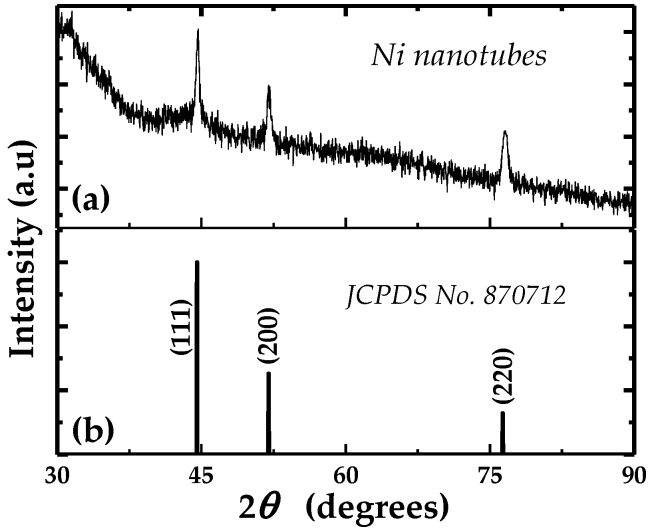
The X-ray diffraction pattern of Ni nanotubes embedded in polycarbonate (PC) template (**a**) with (**b**) JCPDS pattern of the standard Ni is shown for comparison with prepared samples.

Figure 2a shows the X-ray diffraction of Ni nanotubes embedded in the PC template. All the diffraction peaks can be indexed as face-centered cubic Ni (111), (200), and (220), which is in agreement with the standard Ni powder (JCPDS No. 870712) shown in Figure 2b. In the measured XRD data, no significant peak is observed, which indicates that the deposited Ni nanotubes are polycrystalline; however, no preferred orientation is observed in the samples. The polycrystalline nature deduced from XRD analysis is in agreement with TEM results as confirmed by the concentric circular rings that are clearly seen in the SAED pattern shown in the inset of Figure 1c.

**Figure 3:**
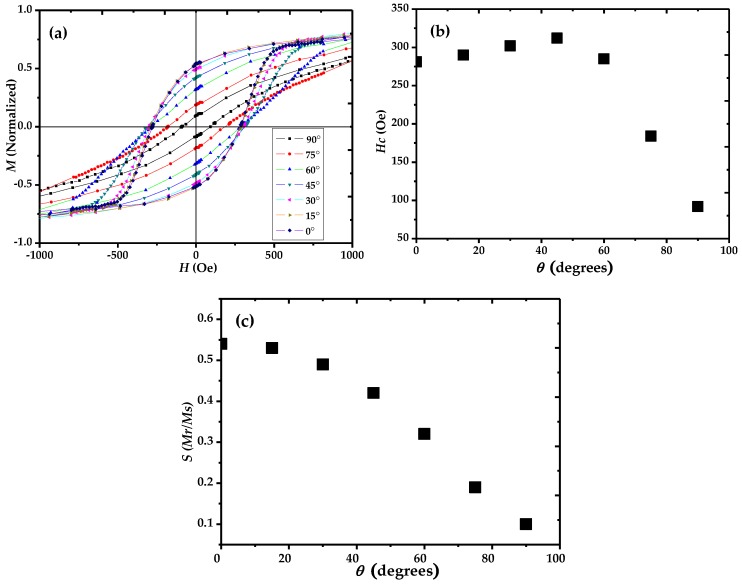
Angular dependence of (**a**) hysteresis loops (**b**) coercivity and (**c**) remanence squareness of Ni nanotube arrays, where θ is the angle between the applied field direction and the tube’s axis.

Figure 3a displays the magnetic hysteresis loops of the Ni nanotubes embedded in the PC templates, which were performed with a VSM with an applied field at different angles (θ) between the field direction and the tube axis ranging from 0° to 90°. Coercivity *H_c_* and remanence squareness *S* (*Mr/Ms*) derived from magnetization curves are presented in Figure 3b,c. In the plot shown in Figure 3b, it is seen that *H_c_* initially increases with increasing angles (≤45°), which represents the curling mode of magnetization. However, above this critical angle, the coercivity decreases drastically, indicating that, for large angles (≥45°), the coherent or transversal reversal mode is dominant. From the theoretical simulations of Landeros [27], the transversal reversal mode is observed only for β ≤ 0.33 (β defined as *r*/*R*, where *r* and *R* are the inner and outer radii of nanotube, respectively). The value of β in our experiment was found to be 0.8, which is much larger than 0.33. This indicates that the decrease of coercivity at θ ≥ 45° was attributed to a coherent reversal mode of magnetization. These results imply that there is a transition of magnetization reversal mechanism in Ni nanotube arrays from the curling mode (for small angles) to the coherent mode (for large angles). On the other hand, the remanence squareness value *S* shown in Figure 3c for Ni nanotubes decreases monotonically as the angle increases from 0° to 90°. The squareness is maximum at θ = 0°, which is obvious in this case as the easy axis of magnetization lies along the length of the Ni nanotubes, while the hard axis is pointing perpendicular to the nanotube length.

**Figure 4:**
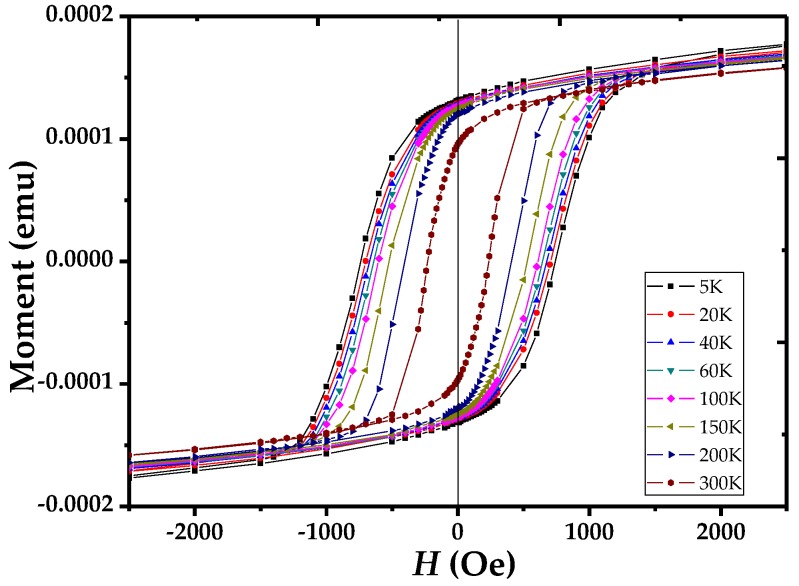
Hysteresis loops of Ni nanotubes taken at 5, 20, 40, 60, 100, 150, 200, and 300 K with the field applied parallel to the tube’s axis.

**Figure 5:**
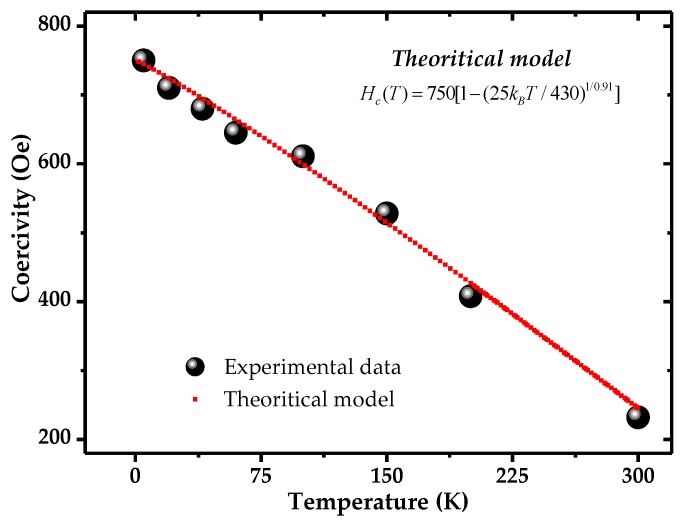
Temperature-dependent coercivity for Ni nanotubes. The red curve shows the fitting curve according to theoretical model discussed in the text.

**Figure 6:**
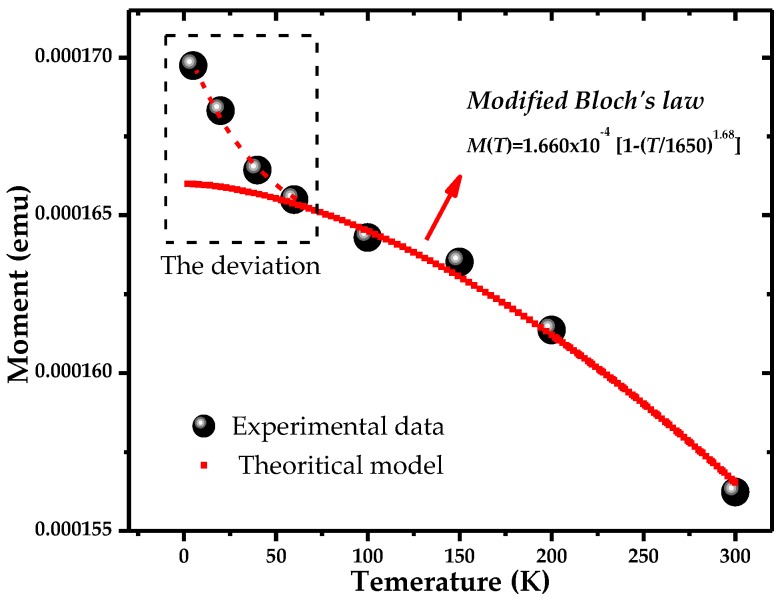
Saturation magnetization as a function of temperature with the red curve representing the modified Bloch’s law.

Figure 4 shows the zero-field-cooled (ZFC) *M*(*H*) loops collected between 5 and 300 K at 2.5 kOe with the direction of the applied field that was fixed along the nanotube (long) axis. The dependence of coercivity and saturation magnetization on temperature was studied and is shown in Figure 5 and Figure 6. From the figures, it can be seen that both coercivity and saturation magnetization increase as the temperature of the nanotubes decreases. To interpret these behaviors at low temperature, we consider the effect of thermal energy on the samples according to Neel’s and Brown’s relation [41,42]:

(1) $$E\;=\;KV\;{\left(1-H/H_{o}\right)}^{n}$$

where *H_o_* is the switching field at *T* = 0 K, *KV* is the anisotropy energy, *E* is the energy barrier at zero field, and *n* is the exponent which is equal to 2 for a system following the Stoner–Wohlfarth model. The relaxation time *τ*, characterizing the process of thermal activation of magnetization over the energy barrier of the system, is given in [2,42]:

(2) $$\tau \;=\;\tau_{o}\;\exp\;(E_{A}/k_{B}T)$$

where τ_o_ is a constant, *E_A_* is the total anisotropy energy, and k_B_ is the Boltzmann constant. Using the above relations, the coercivity relation comes out to be [41]

(3) $$H_{c}(T)\;=\;H_{o}(T)\;[1-{\left(25k_{B}T/KV\right)}^{1/n}]$$

where *H_o_*(*T*) is the zero-temperature coercivity of the system. Now we refer to our data of the temperature dependence of coercivity shown in Figure 5. The red line shows the fit curve according to the above relation using temperature (*T*) as the fitting parameter. This confirms that the coercivity variation with temperature in Ni nanotubes follows the thermal activation model as discussed above.

Magnetization measurements at different temperatures for Ni nanotubes are shown in Figure 6. From the figure, it is evident that saturation magnetization (*M_S_*) increases as the temperature of the samples decreases. For the bulk ferromagnetic system, saturation magnetization below the Curie temperature follows Bloch’s law: *M*(*T*) = *M_o_*[1 − (*T/T_o_*)^α^] [36], where (1/*T_o_*)^α^ is called the Bloch’s constant, which depends on the structure of the material, and α is Bloch’s exponent, which is equal to 1.5 for the bulk materials. However, for the materials at the nanoscale, due to finite size effects, the thermal dependence of magnetization deviates from Bloch’s law, as the magnons with a wavelength larger than the particle dimensions cannot be excited, and a threshold of thermal energy is required to generate spin waves in these materials at the nanoscale. The materials follow the modified Bloch’s law with Bloch’s exponent larger than 1.5.

Therefore, in the case of Ni nanotubes, the spin wave structure is modified in the form of a power law (*T*^α^) with Bloch’s exponent as large as 1.68. In Figure 6, it is evident that saturation magnetization follows the modified Bloch’s law, as shown by the red curve in the figure. Experimental data points follow this law in a high temperature range (60–300 K); however, below 60 K, there seems to be some deviation from this law, which might be the result of reduced atomic coordination at the surface of the nanotubes. The absence of three-dimensional symmetry at the inner and outer surfaces of the nanotubes will change the magnetic spin orientations at their surfaces and therefore it is more likely to alter the magnetic properties in the case of the nanotubes. Similar results have also been reported in the case of magnetic nanoparticles due to the finite size [43,44,45,46]. In our case, the Ni nanotubes have a thin wall with about a 15 nm thickness and have a polycrystalline structure, indicating they are composed of grains with finite size. Attributed to the reduced symmetry of the atomic environment on the inner and outer surfaces, a strong surface anisotropy is induced, which results in the disorder of surface spins in the nanotubes. However, as the temperature decreases, the spins at the inner and outer surfaces become ordered, as the thermal energy is suppressed by the anisotropy energy, which results in the enhancement of the magnetization of the nanotubes. In the temperature range below 60 K, there is an abrupt increase in magnetization of the nanotubes, which shows that the modified Bloch’s law is no more applicable to our system. In the low temperature range, the three-dimensional integration yielding Bloch’s law breaks down for very small wave vectors. This causes the surface spins to be more susceptible to thermal activation, yielding the deviation of experimental data from the modified Bloch’s law at low temperatures [37] and causing increased magnetization of the nanotubes.

## 4. Conclusions

In summary, Ni nanotubes, with an external diameter of 150 nm, a wall thickness of 15 nm, and a length of 1.2 ± 0.3 µm, have been fabricated using a track-etched polycarbonate membrane via a simple electrodeposition technique. A transition between two different modes of magnetization reversal, from the curling mode for small angles to the coherent mode for large angles, was experimentally observed. Temperature magnetic studies indicate that the coercivity of Ni nanotubes follows the thermal activation model at low temperatures, while the saturation magnetization obeys the modified Bloch’s law. The observed deviation of magnetization for lower temperatures (<60 K) is attributed to the finite size effect in the case of Ni nanotubes at the nanoscale.

## References

[1] Cui Y., Zhong Z.H., Wang D.L., Wang W.U., Lieber C.M. (2003). High performance silicon nanowire field effect transistors. Nano Lett..

[2] Maaz K., Duan J.L., Karim S., Chen Y.H., Yao H.J., Mo D., Sun Y.M., Liu J. (2016). Fabrication and low temperature magnetic studies of Ni-Co core-shell nanowires. J. Alloys Compd..

[3] Duan J.L., Lei D.Y., Chen F., Lau S.P., Milne W.I., Toimil-Molares M.E., Trautmann C., Liu J. (2016). Vertically-aligned single-crystal nanocone arrays: Controlled fabrication and enhanced field emission. ACS Appl. Mater. Interfaces.

[4] Liu J., Duan J.L., Toimil-Molares M.E., Karim S., Cornelius T.W., Dobrev D., Yao H.J., Sun Y.M., Hou M.D., Mo D. (2006). Electrochemical fabrication of single-crystalline and polycrystalline Au nanowires: The influence of deposition parameters. Nanotechnology.

[5] Arico A.S., Bruce P., Scrosati B., Tarascon J.M., Van Schalkwijk W. (2005). Nanostructured materials for advanced energy conversion and storage devices. Nat. Mater..

[6] Xia H., Feng J.K., Wang H.L., Lai M.O., Lu L. (2010). MnO_2_ nanotube and nanowire arrays by electrochemical deposition for supercapacitors. J. Power Sources.

[7] Murphy A., McPhillips J., Hendren W., McClatchey C., Atkinson R., Wurtz G., Zayats A.V., Pollard R.J. (2011). The controlled fabrication and geometry tunable optics of gold nanotube arrays. Nanotechnology.

[8] Paramasivam I., Nah Y.C., Das C., Shrestha N.K., Schmuki P. (2010). WO_3_/TiO_2_ nanotubes with strongly enhanced photocatalytic activity. Chem. Eur. J..

[9] Seabra A.B., Duran N. (2013). Biological applications of peptides nanotubes: An overview. Peptides.

[10] Dong Y.B., Xiong C.R., Zhang Y.L., Xing S., Jiang H. (2016). Lithium-titanate-nanotube-supported WO_3_ for enhancing transmittance contrast in electrochromics. Nanotechnology.

[11] Son S.J., Reichel J., He B., Schuchman M., Lee S.B. (2005). Magnetic nanotubes for magnetic-field-assisted bioseparation, biointeraction, and drug delivery. J. Am. Chem. Soc..

[12] Rozman K.Z., Pecko D., Sturm S., Maver U., Nadrah P., Bele M., Kobe S. (2012). Electrochemical synthesis and characterization of Fe_70_Pd_30_ nanotubes for drug-delivery applications. Mater. Chem. Phys..

[13] Yang F., Jin C., Yang D., Jiang Y.J., Li J., Di Y., Hu J.H., Wang C.C., Ni Q.X., Fu D.L. (2011). Magnetic functionalised carbon nanotubes as drug vehicles for cancer lymph node metastasis treatment. Eur. J. Cancer.

[14] Han X.F., Shamaila S., Sharif R., Chen J.Y., Liu H.R., Liu D.P. (2009). Structural and magnetic properties of various ferromagnetic nanotubes. Adv. Mater..

[15] Rohan J.F., Casey D.P., Ahern B.M., Rhen F.M.F., Roy S., Fleming D., Lawrence S.E. (2008). Coaxial metal and magnetic alloy nanotubes in polycarbonate templates by electroless deposition. Electrochem. Commun..

[16] Tao F.F., Gao C.L., Xu Z., Xue Z.L. (2010). A facile synthesis method of nickel nanotubes assisted by polyethylene glycol. Polym. Eng. Sci..

[17] Chen Y.H., Duan J.L., Yao H.J., Mo D., Liu T.Q., Wang T.S., Hou M.D., Sun Y.M., Liu J. (2014). Facile preparation and magnetic properties of Ni nanotubes in polycarbonate ion-track templates. Phys. B Condens. Matter.

[18] Song G.J., Li X.R., Li M.J., Feng S.Y., Zhou C.J. (2016). Fabrication and magnetic properties of Fe, Co and Ni nanotube arrays. Mater. Sci. Forum.

[19] Rozman K.Z., Pecko D., Suhodolcan L., McGuiness P.J., Kobe S. (2011). Electrochemical syntheses of soft and hard magnetic Fe_50_Pd_50_-based nanotubes and their magnetic characterization. J. Alloys Compd..

[20] Kozlovskiy A., Zhanbotin A., Zdorovets M., Manakova I., Ozernoy A., Kadyrzhanov K., Rusakov V. (2015). Study of Ni/Fe nanotube properties. Nucl. Instrum. Methods Phys. Res. Sect. B.

[21] Proenca M.P., Sousa C.T., Ventura J., Vazquez M., Araujo J.P. (2012). Distinguishing nanowire and nanotube formation by the deposition current transients. Nanoscale Res. Lett..

[22] Schaefer S., Felix E.M., Muench F., Antoni M., Lohaus C., Brotz J., Kunz U., Gartner I., Ensinger W. (2016). NiCo nanotubes plated on Pd seeds as a designed magnetically recollectable catalyst with high noble metal utilisation. RSC Adv..

[23] Proenca M.P., Sousa C.T., Ventura J., Araujo J.P., Escrig J., Vazquez M. (2012). Crossover between magnetic reversal modes in ordered Ni and Co nanotube arrays. SPIN World Sci. Publ. Co..

[24] Li X.Z., Wei X.W., Ye Y. (2009). A simple method for forming amorphous rare earth-transition metal alloy nanotube arrays. J. Non-Cryst. Solids.

[25] Li X.Z., Wei X.W., Ye Y. (2009). Template electrodeposition to cobalt-based alloys nanotube arrays. Mater. Lett..

[26] Kamalakar M.V., Raychaudhuri A.K. (2008). A novel method of synthesis of dense arrays of aligned single crystalline copper nanotubes using electrodeposition in the presence of a rotating electric field. Adv. Mater..

[27] Landeros P., Allende S., Escrig J., Salcedo E., Altbir D., Vogel E.E. (2007). Reversal modes in magnetic nanotubes. Appl. Phys. Lett..

[28] Landeros P., Guzman P.R., Soto-Garrido R., Escrig J. (2009). Magnetostatic fields in tubular nanostructures. J. Phys. D Appl. Phys..

[29] Escrig J., Daub M., Landeros P., Nielsch K., Altbir D. (2007). Angular dependence of coercivity in magnetic nanotubes. Nanotechnology.

[30] Escrig J., Landeros P., Altbir D., Vogel E.E., Vargas P. (2007). Phase diagrams of magnetic nanotubes. J. Magn. Magn. Mater..

[31] Zhang X.L., Zhang H.M., Wu T.S., Li Z.Y., Zhang Z.J., Sun H.Y. (2013). Comparative study in fabrication and magnetic properties of FeNi alloy nanowires and nanotubes. J. Magn. Magn. Mater..

[32] Escrig J., Bachmann J., Jing J., Daub M., Altbir D., Nielsch K. (2008). Crossover between two different magnetization reversal modes in arrays of iron oxide nanotubes. Phys. Rev. B.

[33] Proenca M.P., Sousa C.T., Escrig J., Ventura J., Vazquez M., Araujo J.P. (2013). Magnetic interactions and reversal mechanisms in Co nanowire and nanotube arrays. J. Appl. Phys..

[34] Ahmad N., Chen J.Y., Zhou W.P., Liu D.P., Han X.F. (2011). Magnetoelastic anisotropy induced effects on field and temperature dependent magnetization reversal of Ni nanowires and nanotubes. J. Supercond. Nov. Magn..

[35] Ahmad N., Chen J.Y., Iqbal J., Wang W.X., Zhou W.P., Han X.F. (2011). Temperature dependent magnetic properties of Co nanowires and nanotubes prepared by electrodeposition method. J. Appl. Phys..

[36] Bloch F. (1930). On the theory of ferromagnetism. Z. Phys..

[37] Hussain M., Khan M., Sun H.Y., Nairan A., Karim S., Nisar A., Maqbool M., Ahmad M. (2015). Fabrication and temperature dependent magnetic properties of Ni–Cu–Co composite nanowires. Phys. B Condens. Matter.

[38] Kneller E.F., Luborsky F.E. (1963). Particle size dependence of coercivity and remanence of single-domain particles. J. Appl. Phys..

[39] Batlle X., Delmuro M.G., Tejada J., Pfeiffer H., Gornert P., Sinn E. (1993). Magnetic study of M-type doped barium ferrite nanocrystalline powders. J. Appl. Phys..

[40] Maaz K., Usman M., Karim S., Mumtaz A., Hasanain S.K., Bertino M.F. (2009). Magnetic response of core-shell cobalt ferrite nanoparticles at low temperature. J. Appl. Phys..

[41] Brown W.F. (1963). Thermal fluctuation of a single-domain particle. Phys. Rev..

[42] Neel L. (1949). Théorie du traînage mécanique des ferromagnétiques en grains fins avec applications aux terres cuites. Ann. Geofis..

[43] Weller D., Alvarado S.F., Gudat W., Schroder K., Campagna M. (1985). Observation of surface-enhanced magnetic order and magnetic surface reconstruction on Gd (0001). Phys. Rev. Lett..

[44] Kodama R.H., Berkowitz A.E., McNiff E.J., Foner S. (1996). Surface spin disorder in NiFe_2_O_4_ nanoparticles. Phys. Rev. Lett..

[45] Garanin D.A., Kachkachi H. (2003). Surface contribution to the anisotropy of magnetic nanoparticles. Phys. Rev. Lett..

[46] Wang D., Gong M.L. (2011). Surface and shape anisotropy effects in LaFeO_3_ nanoparticles. J. Appl. Phys..

